# Effects of traditional Chinese exercises on post-stroke depression: a meta-analysis of randomized controlled trials

**DOI:** 10.3389/fpubh.2025.1570878

**Published:** 2025-05-12

**Authors:** Yan Li, Xiaomei Li, Jingwen Huang, Hejia Cai

**Affiliations:** ^1^College of Physical Education and Health, Guangxi Normal University, Guilin, China; ^2^Department of Physical Education, Guilin University of Aerospace Technology, Guilin, China; ^3^Guilin Municipal Hospital of Traditional Chinese Medicine, Guilin, China; ^4^Outdoor Sports Academy, Guilin Tourism University, Guilin, China

**Keywords:** post-stroke depression, Taiji, Baduanjin, meta-analysis, traditional Chinese exercises, non-pharmacological therapies

## Abstract

Post-stroke depression (PSD) is a common complication that worsens neurological recovery and increases mortality among stroke survivors, creating a significant burden on patients and families. While exercise interventions are known to alleviate depressive symptoms in mild stroke cases, the effects of traditional Chinese exercises on PSD have not been systematically reviewed. This study aimed to evaluate the clinical efficacy of traditional Chinese exercises in treating PSD. A comprehensive search of eight Chinese and English databases identified randomized controlled trials (RCTs) published up to July 2024. Data were independently screened, extracted, and analyzed using RevMan 5.3 and Stata 17, with heterogeneity assessed through sensitivity, subgroup, and meta-regression analyses. Ten RCTs involving 627 participants were included. Meta-analysis revealed that traditional Chinese exercises significantly reduced depressive symptoms and improved daily living abilities in PSD patients. Specifically, HAMD scores decreased [SMD = −1.40, 95% CI (−1.88, −0.92), *p* < 0.00001], treatment efficacy improved [OR = 3.74, 95% CI (1.69, 8.28), *p* = 0.001], FMA scores increased [MD = 6.22, 95% CI (4.12, 8.32), *p* < 0.00001], and BI levels rose [MD = 4.95, 95% CI (2.96, 6.93), *p* < 0.00001]. In conclusion, traditional Chinese exercises offer significant benefits in treating PSD, demonstrating both efficacy and safety.

**Systematic review registration:** https://www.crd.york.ac.uk/PROSPERO/view/CRD42024574791.

## 1 Introduction

Stroke stands as the leading cause of mortality and disability among adults in China, with its incidence and patient numbers rising annually, driven by the intensifying trend of population aging, currently at approximately 10.7% (1). Post-stroke depression (PSD) is one of the most common and severe complications following a stroke, manifesting as an affective disorder that may emerge within days, weeks, or months after the event. Clinically, PSD is characterized by depressed mood, cognitive decline, and diminished physical activity (2). Statistics reveal that the incidence of depression during the first year post-stroke ranges from 29 to 33%, with a cumulative incidence reaching 39 to 52% within 5 years (3). Patients suffering from PSD experience slower recovery rates, decreased quality of life, and an elevated risk of suicide. Furthermore, PSD often leaves significant motor, emotional, and cognitive impairments, contributing to low treatment adherence and imposing substantial economic burdens on both families and society (4). The pathophysiological mechanisms of PSD are complex, closely linked to the functional deficits and neurochemical changes secondary to brain injury (5). However, the precise pathogenesis remains unclear, presenting significant challenges for clinical treatment.

Currently, numerous treatment options for PSD are available, categorized broadly into pharmacological and non-pharmacological approaches (6). However, pharmacotherapy is often accompanied by serious side effects. For instance, selective serotonin reuptake inhibitors (SSRIs) are widely recognized as the first-line treatment for PSD (2), but they may significantly increase the risk of adverse events such as seizures, falls, and delirium (2). Additionally, late-stage PSD patients often exhibit poor tolerance to antidepressants, highlighting a critical limitation of pharmacotherapy (7). Thus, the quest for an effective, safe, and easily implementable non-pharmacological treatment becomes imperative.

Exercise is one of the most effective means of preventing and treating stroke-related disorders and their complications while promoting overall physical and mental health (8, 9). Extensive research demonstrates that exercise significantly improves depressive symptoms, cognitive function, and daily living abilities in PSD patients (10–12). Chinese traditional exercises (TCEs) such as Tai Chi, Baduanjin Qigong, and Liuzijue, which integrate physical activity with breathing regulation, emphasize the harmony of body and mind, offering remarkable benefits in alleviating depression and ameliorating cognitive impairments (13, 14). Clinical decision support systems (CDSS) offer evidence-based guidance to clinicians, striving to improve patient treatment outcomes (15). However, existing meta-analyses on exercise interventions for PSD are scarce, primarily focusing on aerobic exercise, home-based rehabilitation, and virtual reality training. Therefore, this study aims to systematically evaluate the therapeutic effects of Chinese traditional exercises on PSD, exploring their role in symptom improvement, safety, and efficacy, to provide evidence-based support for clinical practice.

## 2 Materials and methods

### 2.1 Protocol and registration

This study is registered on PROSPERO with registration number CRD42024574791. The study protocol adheres strictly to the PRISMA guidelines (Preferred Reporting Items for Systematic Reviews and Meta-Analyses).

### 2.2 Search strategy

Following the determination of the final search terms, a comprehensive literature search was conducted across eight databases: PubMed, Cochrane Library, Web of Science, Embase, China Biomedical Literature Database (CBM), China National Knowledge Infrastructure (CNKI), Wanfang Database, and China Science and Technology Journal Database (VIP). The search covered the period from the inception of each database up to July 2024, without any restrictions on publication date. The search strategy combined MeSH terms and free-text keywords, with primary search terms including “post-stroke depression,” “traditional Chinese exercise,” and “randomized controlled trial.” Detailed search strategies for each database are provided in Supplementary Table 1.

### 2.3 Inclusion and exclusion criteria

The inclusion criteria, based on the PICO framework, are as follows:

① Population: Patients with post-stroke depression (PSD) meeting the diagnostic criteria.
② Intervention: The experimental group includes TCE such as Tai Chi, Baduanjin, Yijinjing, and Wuqinxi, while the control group receives standard care, health education, or routine treatment.
③ Outcome: Primary outcomes include the efficacy of TCE in alleviating depression, assessed using the Hamilton Depression Rating Scale (HAMD) scores and Overall Efficacy Rate. Secondary outcomes include FMA (Fugl-Meyer motor function assessment) and BI (Breteau Index) scores.
④ Study Design: Only randomized controlled trials (RCTs) were included.

### 2.4 Exclusion criteria

① Dissertations, conference proceedings, studies without full text access, or those with missing data.
② Patients without PSD.
③ Duplicate publications.
④ Studies with outcome measures not aligned with the objectives of this research.
⑤ Studies involving subjects with comorbid malignancies or severe hepatic or renal dysfunction.

### 2.5 Study selection

All retrieved records were imported into Endnote20 for automatic and manual deduplication. Subsequently, two researchers independently screened the titles, abstracts, and full texts based on the inclusion and exclusion criteria. Discrepancies were resolved through discussion or consultation with a third researcher. If necessary, the original authors were contacted by email or phone for additional information.

### 2.6 Data extraction

The extracted data included: (1) basic information of the included studies, such as title, first author, and publication date; (2) baseline characteristics of the study population, including mean age and sample size; (3) detailed descriptions of the intervention and its duration; (4) outcome measures.

### 2.7 Quality assessment

Given that all included studies were RCTs, the Cochrane Risk of Bias Tool (version 5.1.0) was employed for quality assessment (16). Two researchers with relevant expertise independently assessed the risk of bias, focusing on seven domains: random sequence generation, allocation concealment, blinding of participants and personnel, blinding of outcome assessment, completeness of outcome data, selective reporting, and other biases. Each domain was rated as low risk, high risk, or unclear risk.

### 2.8 Data analysis

Meta-analysis was conducted using Revman 5.3 and Stata 17 software. Odds ratios (OR) with 95% confidence intervals (CI) were calculated for dichotomous outcomes, while mean differences (MD) were used for continuous outcomes with consistent units; otherwise, standardized mean differences (SMD) were employed. Heterogeneity was assessed using the *χ*^2^ test (with *α* = 0.1) and quantified with the *I*^2^ statistic. A fixed-effect model was used when *I*^2^ < 50%, and a random-effects model was applied when *I*^2^ ≥ 50%. Statistical significance was set at *α* = 0.05. Subgroup analyses were conducted to explore sources of heterogeneity, and sensitivity analyses were performed by excluding individual studies to assess the robustness of the results. To address publication bias, both qualitative (funnel plot) and quantitative (Egger’s test) methods were used.

## 3 Results

### 3.1 Search results

**Figure 1:**
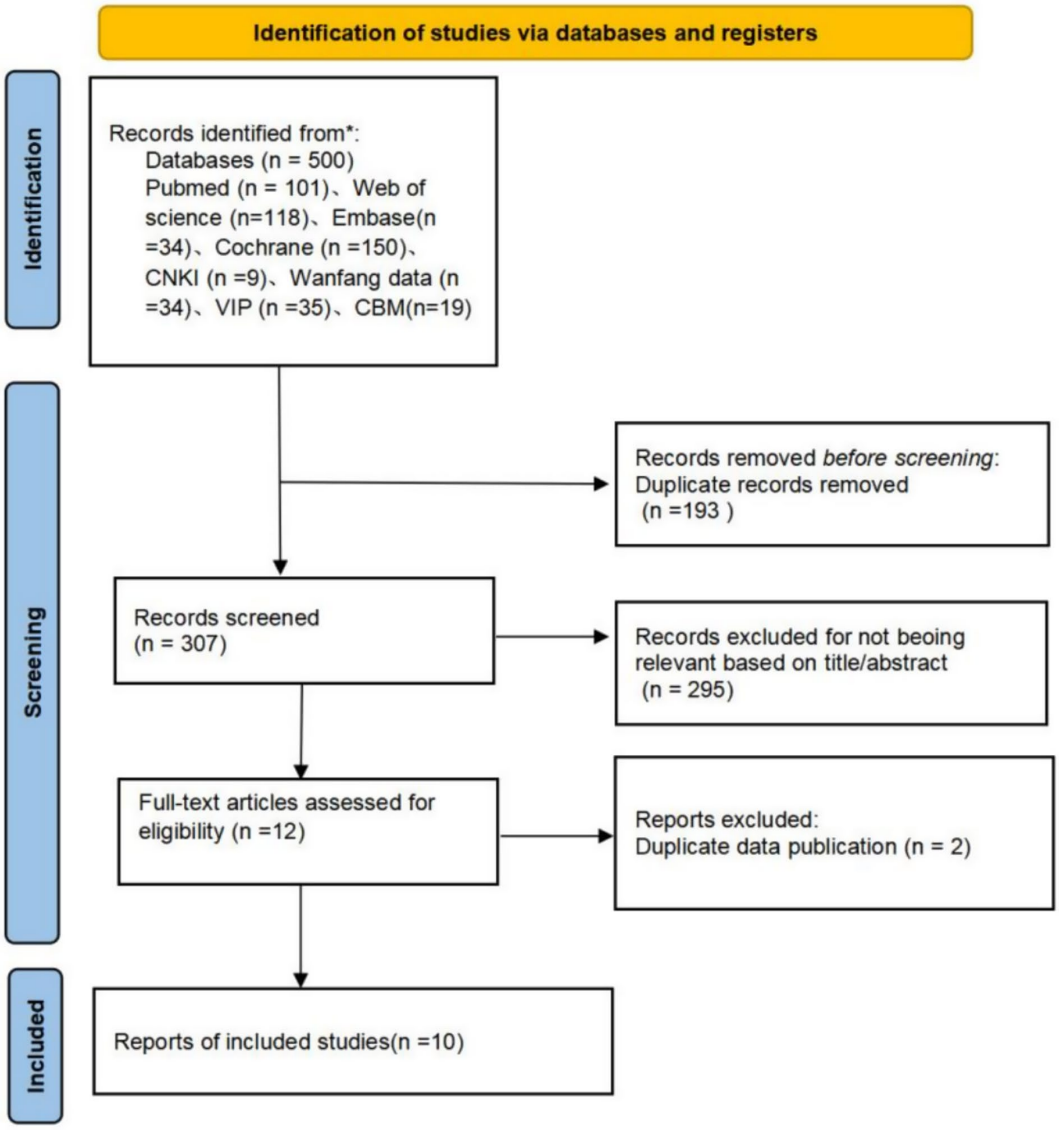
PRISMA flowchart of article inclusion.

The systematic search yielded 500 studies. After title, abstract, and full-text screening according to the inclusion and exclusion criteria, 490 studies were excluded, leaving 10 RCTs for inclusion (17–26). The study selection process is depicted in Figure 1.

### 3.2 Characteristics of included studies

The 10 included studies (17–26) involved a total of 627 participants, with 317 in the intervention group and 310 in the control group. Seven studies (17–22, 26) utilized the HAMD-24 as an outcome measure, three studies (23–25) used the HAMD-17, three studies (18–20) reported response rates, six studies (17, 18, 20, 22, 23, 26) reported FMA scores, and three studies (17, 23, 26) reported BI scores. Table 1 summarizes the baseline characteristics of the included studies.

**Table 1:** Characteristics of 10 eligible articles.

Study	Research object		Intervention measure			Outcome index
	Age	sample number	Mode of movement	Exercise time and frequency	Intervention cycle	
Li et al. (19)	T:38 ~ 76	30	Tai ji + Routine care	30 min, twice a week	5 weeks	①
	C:38–76	30	Routine care			
Wang et al. (24)	T:55.8 ± 3.54	36	Tai ji	Twice a week	3 Months	①
	C:51. 2 ± 7. 8	33	Medical treatement			
Zhao et al. (26)	T:53.85 ± 11.69	30	Conventional rehabilitation therapy + Tai Chi	30 min, 5 days a week	8 weeks	①, ②, ③, ④
	C:51.38 ± 14.83	30	Conventional rehabilitation therapy			
Li et al. (18)	T:71.03 ± 8.21	30	Tai ji + Drug Treatment	Once a day for 1 h	12 weeks	①, ②, ③
	C:71.06 ± 8.33	30	Medical treatement			
Du et al. (17)	T:63.67 ± 6.97	27	Traditional exercises + Conventional treatment	20 min, 5 days a week	8 weeks	①, ③, ④
	C:65.24 ± 7.68	26	Conventional treatment			
Liu et al. (20)	T:57.58 ± 5.71	30	Ba Duan Jin + medical treatement	45 min, 3 days a week	4 weeks	①, ②, ③
	C:56.85 ± 7.47	30	Medical treatement			
Sun et al. (21)	T:62.03 ± 7.37	30	Yi Jin Jing + Conventional treatment	Once a day for 1 h	3 weeks	①
	C:65.23 ± 6.29	30	Conventional treatment			
Zhang and Chen (25)	T:45.39 ± 8. 61	28	Six-Character Formula + Nursing interventions	Once a day for 20 ~ 25 min	4 weeks	①
	C:43.12 ± 7. 35	29	Nursing interventions			
Tang et al. (22)	T:57.18 ± 7.71	30	Ba Duan Jin + medical treatement	2 times a day, 15 min each time, at least 6 times a week	8 weeks	①, ③
	C:57.14 ± 7.68	30	medical treatement			
Wang et al. (23)	T:56.86 ± 3.37	40	Tai ji + Conventional rehabilitation therapy	30 min, 5 days a week	2 Months	①, ③, ④
	C:56.35 ± 3.35	40	Conventional rehabilitation therapy			

① = HAMD; ② = Total effectiveness rate; ③ = FMA; ④ = BI; T, Experimental group; C, Control group.

### 3.3 Quality assessment

**Figure 2:**
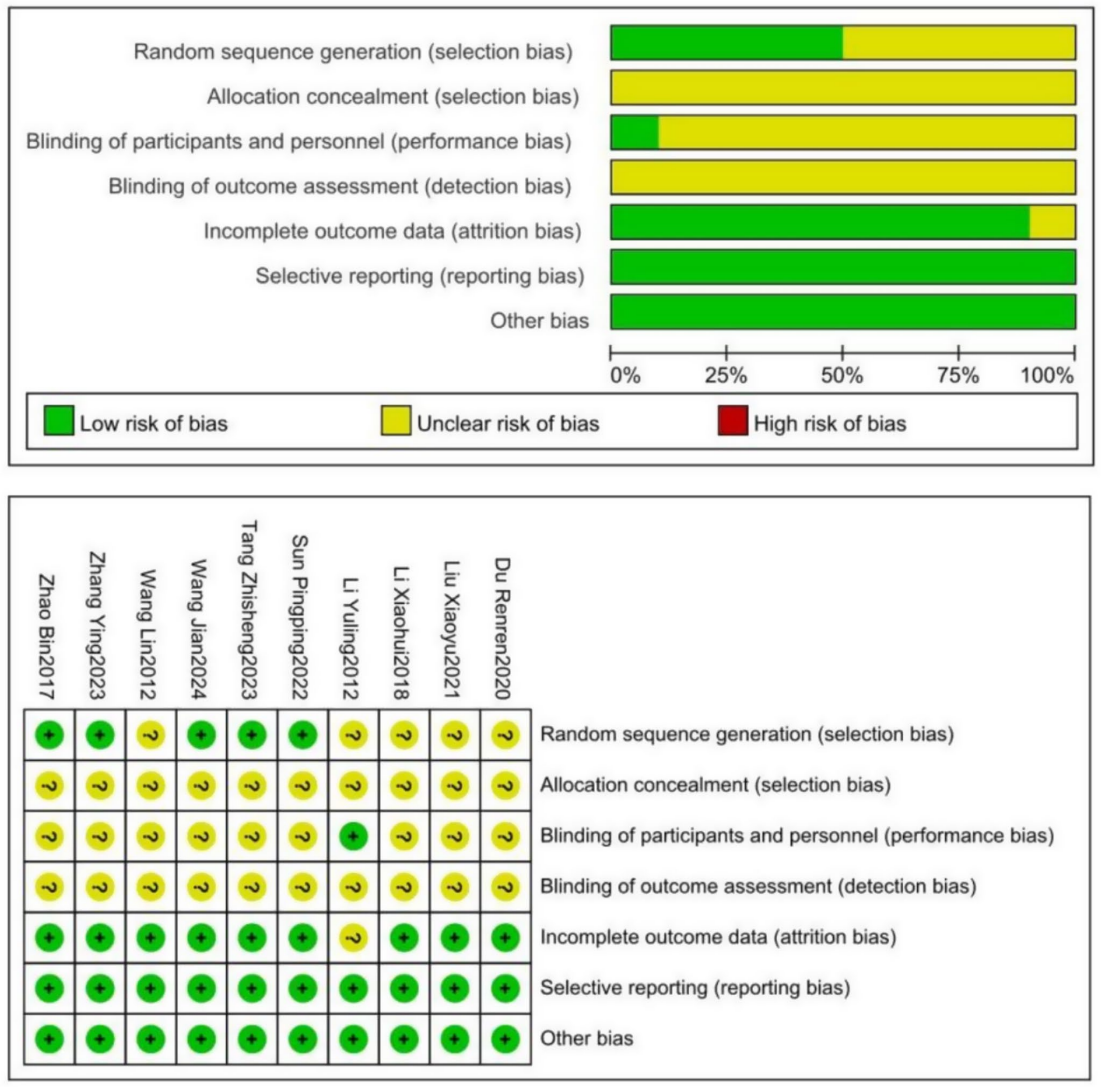
Schematic of cochrane bias risk assessment.

Among the 10 included studies, five studies (21–23, 25, 26) explicitly reported the method of random sequence generation, while the others vaguely mentioned “randomization” without specifying the method. Except for one study (19), none described allocation concealment or blinding procedures, resulting in unclear risk of bias. One study (17) reported a small number of dropouts with reasons provided, while the remaining studies had complete data and were rated as low risk. No other biases were reported in the included studies. The methodological quality assessment of the studies is presented in Figure 2.

### 3.4 Meta-analysis results

#### 3.4.1 HAMD

**Figure 3:**
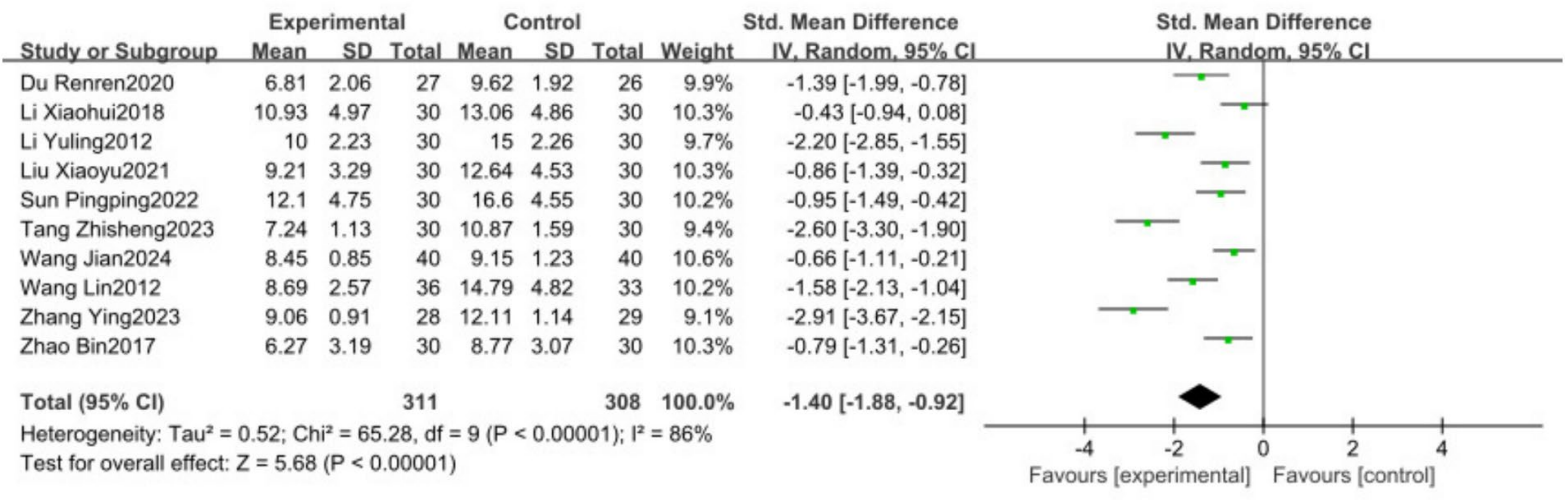
Forest plots showing the effect of TCP on depression outcomes.

A total of 10 studies (17–26) encompassing 619 patients reported HAMD scores and were included in the meta-analysis. The results indicated substantial heterogeneity (*I*^2^ = 86%). A random-effects model revealed that traditional Chinese exercise significantly reduced HAMD levels in patients with PSD [SMD = −1.40, 95% CI (−1.88, −0.92), *p* < 0.00001], as shown in Figure 3.

#### 3.4.2 Overall efficacy rate

**Figure 4:**
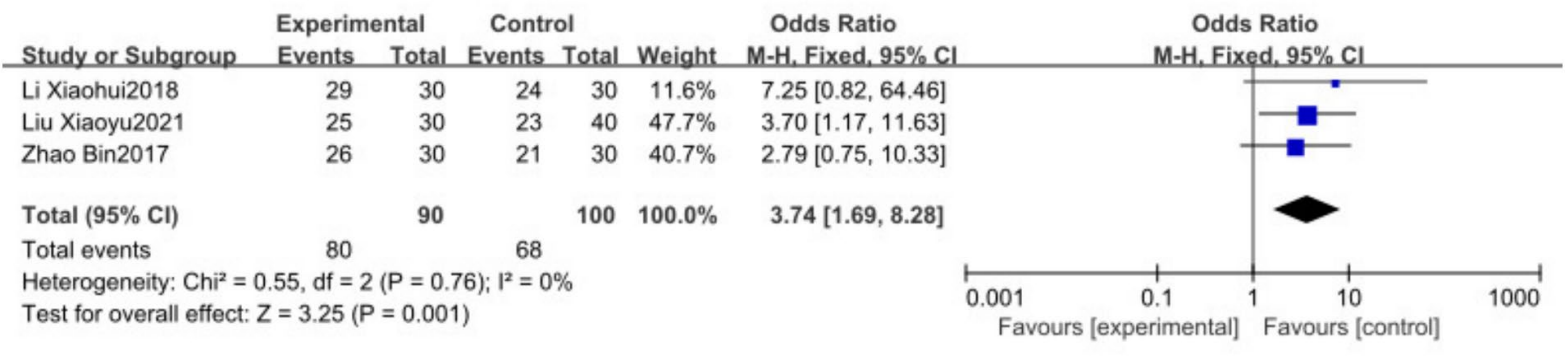
Forest plots showing the effect of TCP on Overall Efficacy Rate outcomes.

Three studies (18, 20, 26) involving 270 patients reported the overall efficacy rate and were included in the meta-analysis. The heterogeneity analysis showed no significant heterogeneity among the studies (*I*^2^ = 0, *p* = 0.76). Therefore, a fixed-effects model indicated that the overall efficacy rate in the experimental group was significantly higher than that in the control group [OR = 3.74, 95% CI (1.69, 8.28), *p* = 0.001], as illustrated in Figure 4.

#### 3.4.3 FMA

**Figure 5:**
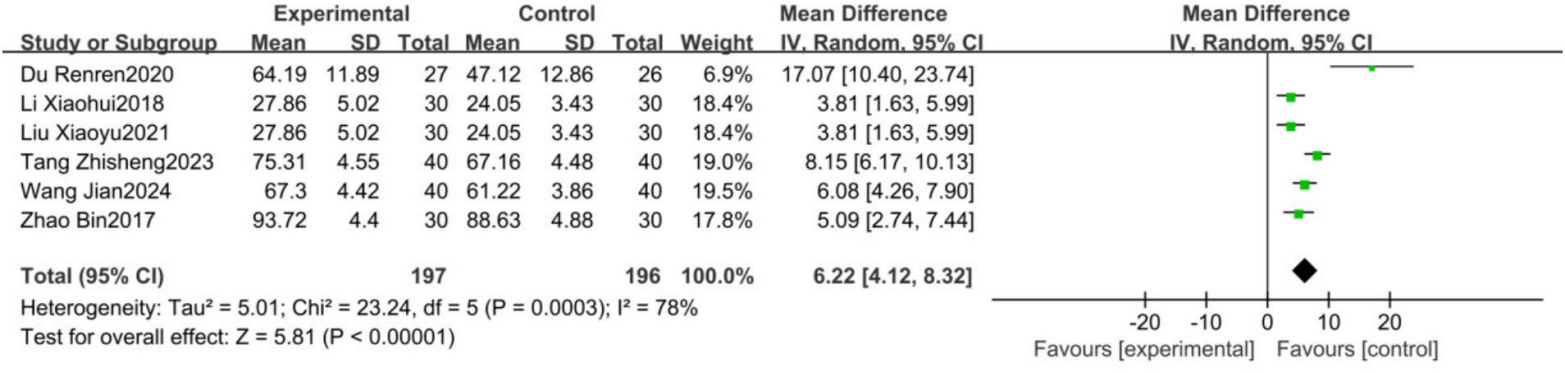
Forest plots showing the effect of FMA on depression outcomes.

Six studies (17, 18, 20, 22, 23, 26) with a total of 373 patients reported FMA scores and were included in the meta-analysis. The analysis revealed considerable heterogeneity (*I*^2^ = 78%). A random-effects model showed that traditional Chinese exercise significantly improved FMA levels in PSD patients [MD = 6.22, 95% CI (4.12, 8.32), *p* < 0.00001], as depicted in Figure 5.

#### 3.4.4 BI

**Figure 6:**
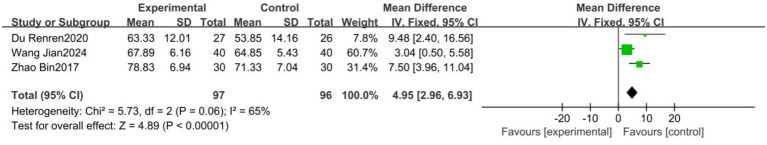
Forest plots showing the effect of BI on depression outcomes.

Three studies (17, 23, 26) involving 193 patients reported BI scores and were included in the meta-analysis. The analysis revealed significant heterogeneity (*I*^2^ = 65%). A random-effects model demonstrated that traditional Chinese exercise significantly enhanced BI levels in PSD patients [MD = 4.95, 95% CI (2.96, 6.93), *p* < 0.00001], as presented in Figure 6.

### 3.5 Subgroup analysis

**Figure 7:**
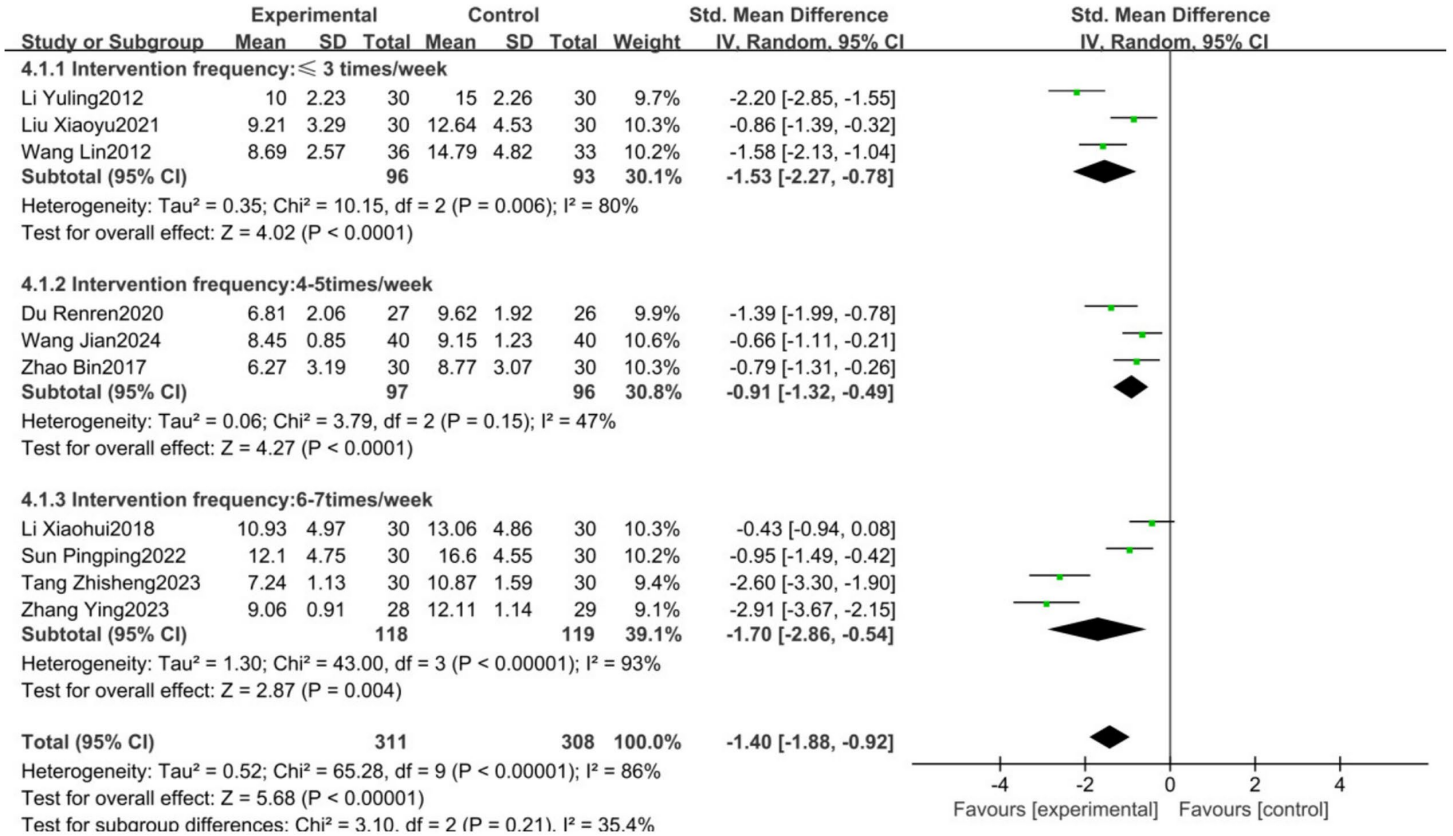
Forest plot showing the effect of different training frequencies on HAMD results.

**Figure 8:**
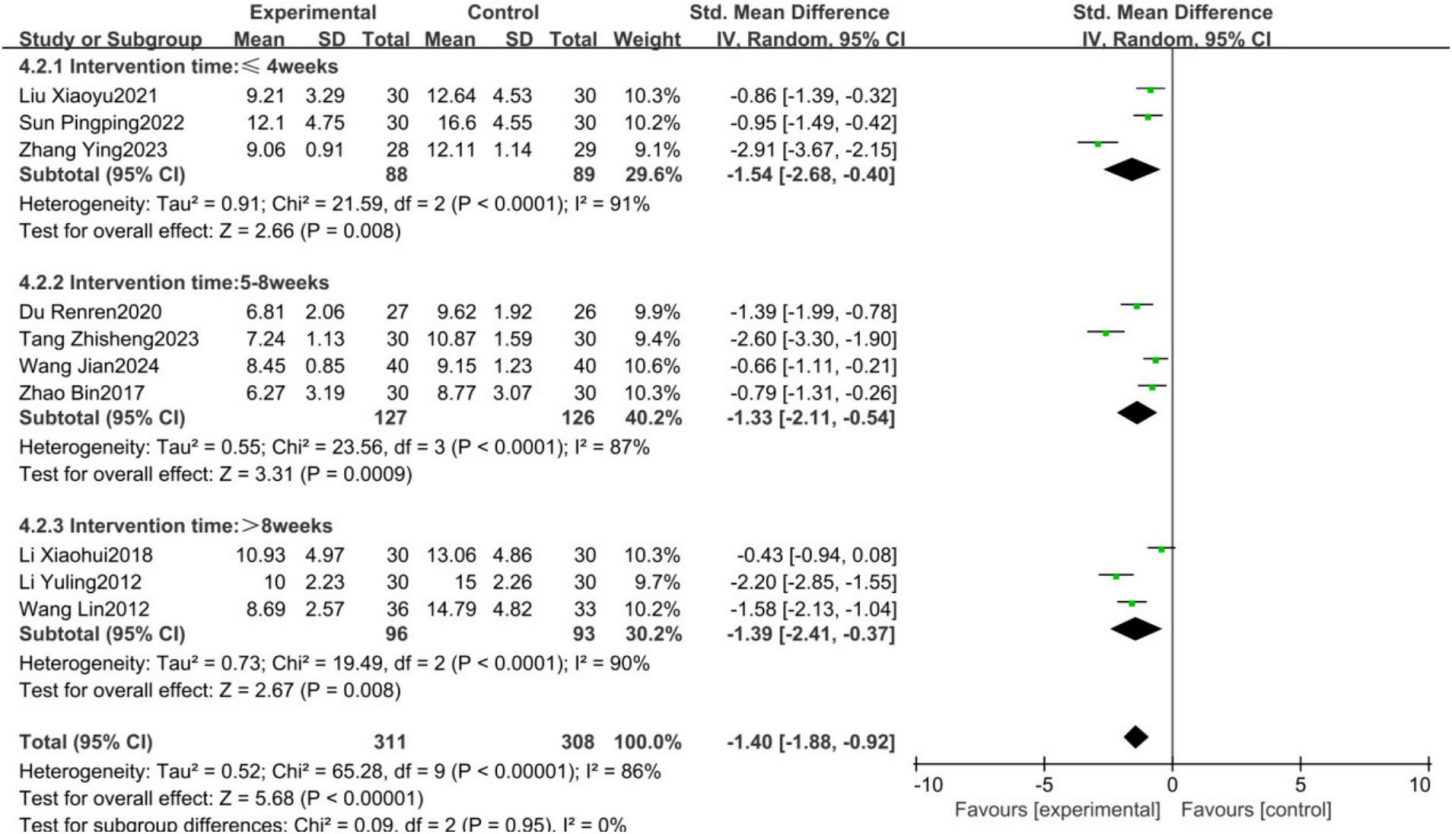
Forest plot showing the effect of different training durations on HAMD results.

**Figure 9:**
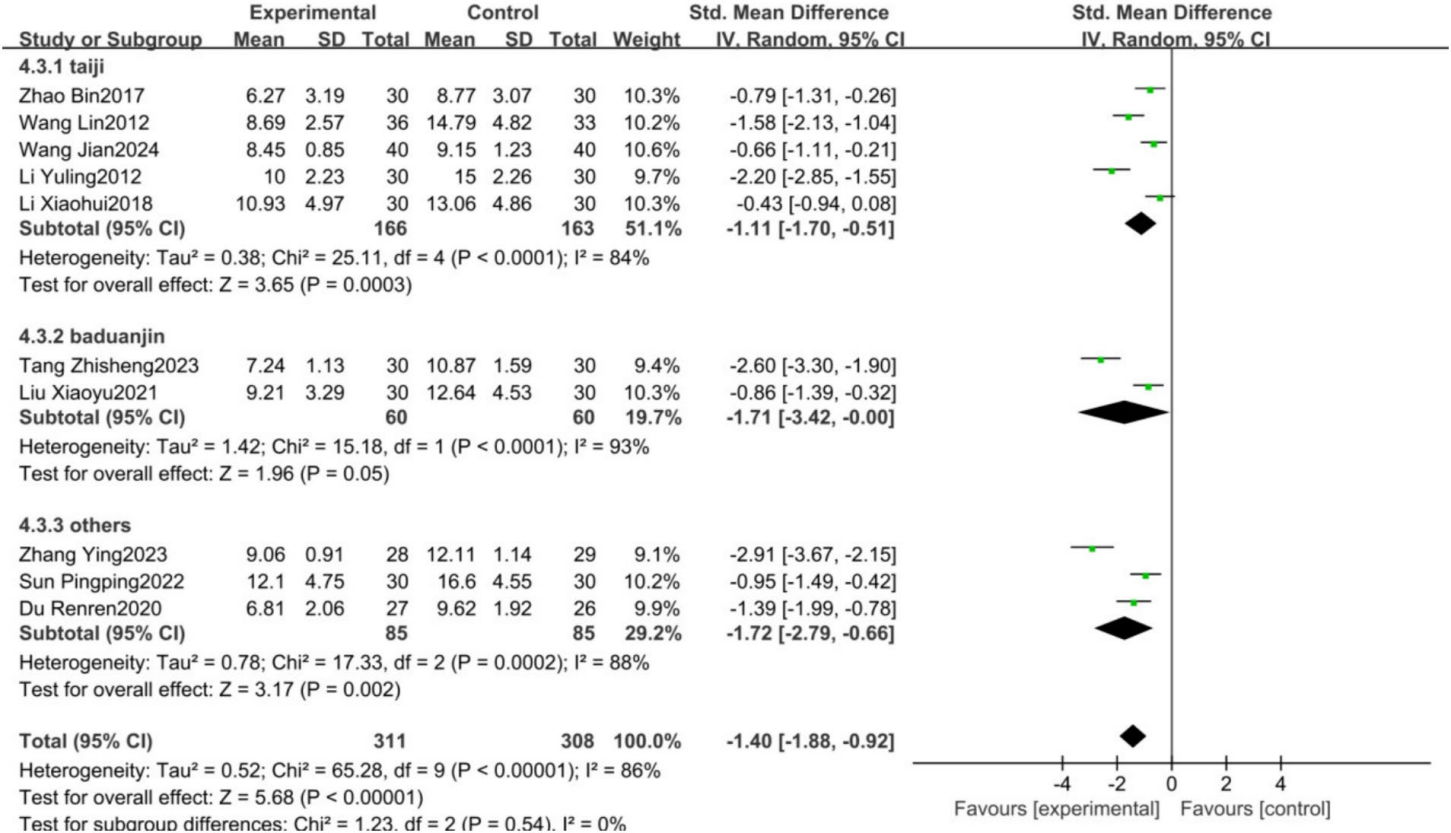
Forest plot showing the effect of different training programs on HAMD Results.

Given the high heterogeneity observed in the primary HAMD outcome, subgroup analyses were conducted based on the frequency of exercise intervention (3 times/week; 4–5 times/week; 6–7 times/week), intervention duration (short-term: ≤4 weeks; mid-term: 5–8 weeks; long-term: >8 weeks), and type of exercise (Tai Chi, Baduanjin, others). The results showed no statistical heterogeneity among subgroups with different intervention frequencies (*p* = 0.21, *I*^2^ = 35.4%), with the 4–5 times/week subgroup demonstrating the lowest heterogeneity (*p* = 0.15, *I*^2^ = 47%), suggesting that intervention frequency may be a source of heterogeneity (Figure 7). There was no statistical heterogeneity among subgroups with different intervention durations (*p* = 0.95, *I*^2^ = 0%); however, substantial heterogeneity persisted among subgroups, indicating that intervention duration is not the primary source of heterogeneity (Figure 8). Similarly, no statistical heterogeneity was found among subgroups with different types of exercise intervention (*p* = 0.54, *I*^2^ = 0%), yet significant heterogeneity remained, implying that the type of exercise intervention is also not a major source of heterogeneity (Figure 9).

### 3.6 Sensitivity analysis

Sensitivity analysis was conducted to assess the robustness of the pooled data and further explore sources of heterogeneity. By sequentially excluding individual studies, it was found that excluding the study by Wang Jian (23) resulted in a pooled BI effect of [MD = 7.90, 95% CI (4.73, 11.06), *p* < 0.00001] with *I*^2^ decreasing from 65 to 0%. No significant changes were observed in the results of other outcomes, confirming the stability and reliability of the study findings.

### 3.7 Adverse events

All 10 studies included in this analysis reported no adverse events or safety concerns, indicating that traditional Chinese exercises are both safe and reliable.

### 3.8 Publication bias

**Figure 10:**
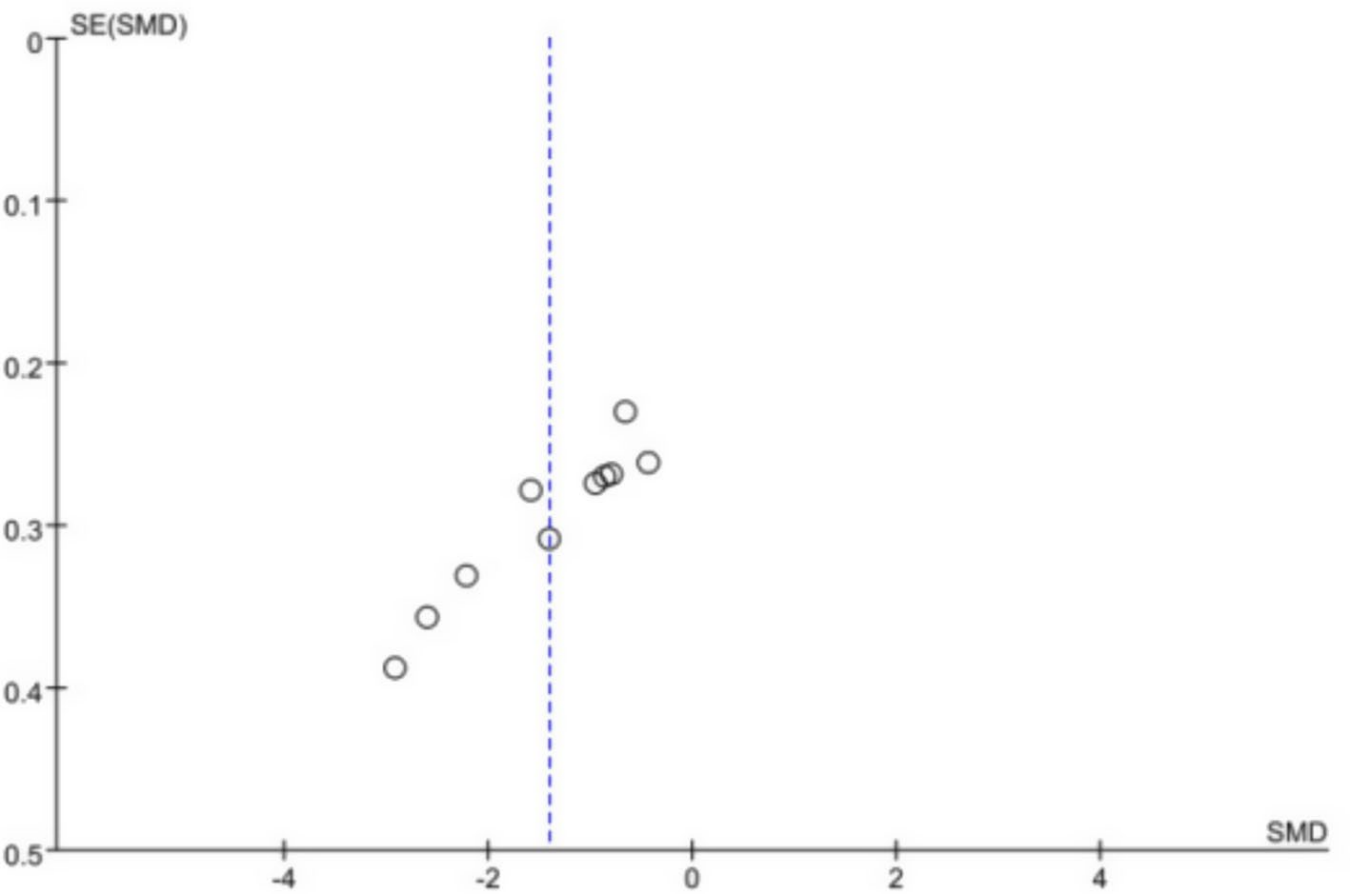
Funnel plot of all included studies for HAMD.

Due to the limited number of included studies, publication bias was assessed only for the HAMD outcome. Funnel plot and Egger’s test analyses indicated that the 10 scatter points were distributed on both sides of the centerline, though not perfectly symmetrical (Figure 10). Additionally, Egger’s test showed a result of *p* < 0.05 (see Supplementary Figure 1), suggesting a potential publication bias among the included studies.

## 4 Discussion

Based on the existing evidence, the primary exercise therapies for post-stroke depression focus predominantly on aerobic and resistance training. Numerous studies have demonstrated that these therapies significantly improve the depressive symptoms, cognitive function, and activities of daily living in stroke patients (27–29). However, for older adults patients with frailty and chronic conditions, the intensity and volume of aerobic and resistance exercises can be challenging to manage. Additionally, the monotonous nature of these exercises often leads to patient dropout.

The included studies exhibited notable deficiencies in the implementation of allocation concealment, a critical component of research design. Allocation concealment ensures that both implementers and participants remain unaware of intervention assignments. Like the generation of random sequences, proper implementation of both measures can effectively reduce selection bias. Without adequate allocation concealment, even well-executed randomization may lead to an overestimation of treatment effects. Researchers are advised to prioritize the implementation of allocation concealment in clinical studies and provide accurate, detailed reporting of the methods employed. Specific techniques include centralized telephone or fax randomization, as well as sequentially numbered, sealed, opaque envelopes to minimize allocation bias.

The necessity of blinding primarily lies in reducing performance bias and detection bias during research. However, not all studies can achieve double-or triple-blinding, which may explain why many Chinese studies omit reporting blinding procedures. In such cases, the assessment of bias risk related to blinding should be considered on a case-by-case basis: if objective outcome measures are used, blinding may have minimal impact on results; conversely, subjective outcome measures may be more susceptible to bias.

Traditional Chinese exercises, such as Tai Chi, Baduanjin, Yijinjing, and Wuqinxi, are distinctive forms of physical activity within traditional Chinese medicine. These practices emphasize mental focus, slow movements, and low intensity, aiming to harmonize the body and mind, regulate qi and blood circulation, prevent disease, and promote health by integrating physical training with breathing and mental exercises (30). Compared to other forms of exercise, traditional practices are not bound by time or space, are simple, safe, and effective, and possess a moderate intensity, making them particularly suitable for the rehabilitation of patients with post-stroke depression. Tai Chi, in particular, has been proven to be highly effective in preventing depression in the older adults, with no side effects and easy acceptance (31).

This meta-analysis included 10 RCTs involving 627 patients with post-stroke depression. The results indicated that traditional Chinese exercises significantly reduced depressive symptoms, improved physical and daily living abilities, and had a high safety profile compared to the control group. The meta-analysis results for all indicators demonstrated large effect sizes, suggesting that traditional Chinese exercises are highly effective in the intervention for patients with post-stroke depression. As a regular, low-to moderate-intensity aerobic exercise, traditional Chinese exercises help enhance cellular and tissue metabolism, increase venous return, and activate the prefrontal cortex (32). Furthermore, traditional Chinese exercises have shown significant intervention effects on general depression, particularly in the older adults, likely due to the alignment of these exercises with the slower pace and moderate intensity that are more suitable for the physical fitness levels of older adults (33). The design of traditional Chinese exercises considers the pathways of meridians and acupuncture points, aligning with traditional Chinese medicine’s meridian theory, which posits that maintaining unobstructed meridians contributes to health preservation. During exercise, the coordinated movement of the eyes and body, along with the activity of neck muscles, aids in cerebral blood flow and enhances peripheral nerve responses, thereby alleviating neurological tension and improving mental depression (34) Studies have shown that Tai Chi enhances lower limb muscle strength, improves cardiovascular and respiratory system functions (35, 36) and simultaneously improves flexibility and balance, thereby promoting the ability to perform daily activities. Tai Chi commonly uses 24 forms, while Baduanjin consists of only 8 forms, making it more suitable for beginners. The Liu Zi Jue breathing exercise can enhance memory and responsiveness in stroke patients while promoting emotional calmness (37). Additionally, studies have found that Baduanjin and Yijinjing, which emphasize muscle relaxation, mental relaxation, and breath regulation, significantly improve cardiovascular health, flexibility, balance, executive function, and lumbar strength over long-term practice, as well as physiological and biochemical indicators related to mood, such as serotonin, endorphins, and plasma adiponectin (38, 39). However, research on long-term interventions for patients with post-stroke depression remains limited, possibly due to the challenges faced by researchers, including the significant time and financial costs involved. Additionally, the lack of strong motivation from patients themselves may hinder their ability to sustain long-term practice.

Given the significant heterogeneity in some study results, a subgroup analysis was conducted to explore the sources of heterogeneity. The analysis revealed that differences in exercise frequency were the primary contributors to the heterogeneity in combined HAMD results. Furthermore, sensitivity analysis through the sequential exclusion of studies showed that the outcomes for each endpoint remained consistent with those before exclusion, indicating the robustness and reliability of the findings. Inevitably, other confounding factors, such as age and gender (which we are unable to adjust for in the meta-analysis), may exert some influence on the results. We conducted subgroup and sensitivity analyses to explore heterogeneity, but significant variations persisted in key outcomes such as HAMD scores. Factors such as stroke severity, intervention fidelity, or differences in participant motivation may also contribute to heterogeneity and warrant further investigation.

The included studies reported the frequency and duration of exercises but lacked details regarding specific intervention content, delivery methods (e.g., group vs. individual sessions), and instructor qualifications. Although none of the studies documented adverse events, underreporting remains a possibility. Future research should incorporate standardized safety monitoring protocols to ensure comprehensive reporting.

### 4.1 Limitations

First, although both Chinese and English literature were screened, all included studies were in Chinese, likely because exercises such as Tai Chi and Qigong are unique to China. Second, the majority of the included studies were assessed as having a moderate risk of bias. In this review, many RCTs did not report allocation concealment and blinding, leading to unclear risk of bias, which can often result in selective bias during study design and implementation. Finally, this study has a degree of publication bias. Research on the long-term therapeutic effects of traditional Chinese exercise for post-stroke depression remains limited and warrants further exploration. Therefore, future studies should prioritize large-sample, high-quality, multicenter randomized controlled trials (RCTs) with extended follow-up periods and high homogeneity to comprehensively evaluate the benefits and limitations of this intervention, thereby providing more robust evidence for clinical practice.

## 5 Conclusion

This systematic review and meta-analysis found that exercise therapies, including Tai Chi and Qigong, significantly improve depressive symptoms and enhance physical and daily living abilities in patients with post-stroke depression. However, due to the methodological shortcomings of many RCTs, it is challenging to determine the optimal exercise intervention regimen, and the long-term effects of exercise therapies remain unexplored, potentially impacting the study results. Therefore, future high-quality RCTs are recommended to further investigate the long-term impact of exercise therapies on post-stroke depression and to provide evidence for the development of exercise therapy management guidelines for these patients.

## Data Availability

The raw data supporting the conclusions of this article will be made available by the authors, without undue reservation.
