# Supplementary material for: Effects of traditional Chinese exercises on post-stroke depression: a meta-analysis of randomized controlled trials
Source: Front Public Health. 2025 May 12;13:1570878. doi: 10.3389/fpubh.2025.1570878 (PMC12125479; doi:10.3389/fpubh.2025.1570878)
Supplement: Supplementary file 1 [file Table_1.docx]

Supplementary Table 1.Search strategy for each database

| Search engine | Search query | Result |
| --- | --- | --- |
| Pubmed | #1 "mind body therapies"[MeSH Terms]  #2 "tai chi"[Title/Abstract] OR "taiji*"[Title/Abstract] OR "Qigong"[Title/Abstract] OR "Liuzijue"[Title/Abstract] OR "Wuqinxi"[Title/Abstract] OR "Yijinjing"[Title/Abstract] OR "Baduanjin"[Title/Abstract] OR "traditional exercise"[Title/Abstract] OR "traditional chinese"[Title/Abstract] OR "Medicine"[Title/Abstract] OR "chinese traditional exercise"[Title/Abstract] OR "traditional chinese"[Title/Abstract] OR "chinese exercise"[Title/Abstract] OR ("mindbody"[All Fields] AND "exercise"[Title/Abstract]) OR ("mindbody"[All Fields] AND "Therapies"[Title/Abstract])  #3 "post-stroke"[All Fields] AND ("depressive disorder"[MeSH Terms] OR "depression"[MeSH Terms])  #4 "PSD"[Title/Abstract] OR "post stroke depressive"[Title/Abstract] OR "depression after stroke"[Title/Abstract] OR (("depressed"[All Fields] OR "depression"[MeSH Terms] OR "depression"[All Fields] OR "depressions"[All Fields] OR "depression s"[All Fields] OR "depressive disorder"[MeSH Terms] OR ("depressive"[All Fields] AND "disorder"[All Fields]) OR "depressive disorder"[All Fields] OR "depressivity"[All Fields] OR "depressive"[All Fields] OR "depressively"[All Fields] OR "depressiveness"[All Fields] OR "depressives"[All Fields]) AND "in stroke patients"[Title/Abstract]) OR "post stroke depression"[Title/Abstract] OR "post stroke depressive disorder"[Title/Abstract] OR (("depressive disorder"[MeSH Terms] OR ("depressive"[All Fields] AND "disorder"[All Fields]) OR "depressive disorder"[All Fields]) AND "after stroke"[Title/Abstract])  #5 "tai chi"[Title/Abstract] OR "taiji*"[Title/Abstract] OR "Qigong"[Title/Abstract] OR "Liuzijue"[Title/Abstract] OR "Wuqinxi"[Title/Abstract] OR "Yijinjing"[Title/Abstract] OR "Baduanjin"[Title/Abstract] OR "traditional exercise"[Title/Abstract] OR "traditional chinese"[Title/Abstract] OR "Medicine"[Title/Abstract] OR "chinese traditional exercise"[Title/Abstract] OR "traditional chinese"[Title/Abstract] OR "chinese exercise"[Title/Abstract] OR ("mindbody"[All Fields] AND "exercise"[Title/Abstract]) OR ("mindbody"[All Fields] AND "Therapies"[Title/Abstract]) OR "mind body therapies"[MeSH Terms]  #6 ("post-stroke"[All Fields] AND ("depressive disorder"[MeSH Terms] OR "depression"[MeSH Terms])) OR ("PSD"[Title/Abstract] OR "post stroke depressive"[Title/Abstract] OR "depression after stroke"[Title/Abstract] OR (("depressed"[All Fields] OR "depression"[MeSH Terms] OR "depression"[All Fields] OR "depressions"[All Fields] OR "depression s"[All Fields] OR "depressive disorder"[MeSH Terms] OR ("depressive"[All Fields] AND "disorder"[All Fields]) OR "depressive disorder"[All Fields] OR "depressivity"[All Fields] OR "depressive"[All Fields] OR "depressively"[All Fields] OR "depressiveness"[All Fields] OR "depressives"[All Fields]) AND "in stroke patients"[Title/Abstract]) OR "post stroke depression"[Title/Abstract] OR "post stroke depressive disorder"[Title/Abstract] OR (("depressive disorder"[MeSH Terms] OR ("depressive"[All Fields] AND "disorder"[All Fields]) OR "depressive disorder"[All Fields]) AND "after stroke"[Title/Abstract]))  #7 ("tai chi"[Title/Abstract] OR "taiji*"[Title/Abstract] OR "Qigong"[Title/Abstract] OR "Liuzijue"[Title/Abstract] OR "Wuqinxi"[Title/Abstract] OR "Yijinjing"[Title/Abstract] OR "Baduanjin"[Title/Abstract] OR "traditional exercise"[Title/Abstract] OR "traditional chinese"[Title/Abstract] OR "Medicine"[Title/Abstract] OR "chinese traditional exercise"[Title/Abstract] OR "traditional chinese"[Title/Abstract] OR "chinese exercise"[Title/Abstract] OR ("mindbody"[All Fields] AND "exercise"[Title/Abstract]) OR ("mindbody"[All Fields] AND "Therapies"[Title/Abstract]) OR "mind body therapies"[MeSH Terms]) AND (("post-stroke"[All Fields] AND ("depressive disorder"[MeSH Terms] OR "depression"[MeSH Terms])) OR ("PSD"[Title/Abstract] OR "post stroke depressive"[Title/Abstract] OR "depression after stroke"[Title/Abstract] OR (("depressed"[All Fields] OR "depression"[MeSH Terms] OR "depression"[All Fields] OR "depressions"[All Fields] OR "depression s"[All Fields] OR "depressive disorder"[MeSH Terms] OR ("depressive"[All Fields] AND "disorder"[All Fields]) OR "depressive disorder"[All Fields] OR "depressivity"[All Fields] OR "depressive"[All Fields] OR "depressively"[All Fields] OR "depressiveness"[All Fields] OR "depressives"[All Fields]) AND "in stroke patients"[Title/Abstract]) OR "post stroke depression"[Title/Abstract] OR "post stroke depressive disorder"[Title/Abstract] OR (("depressive disorder"[MeSH Terms] OR ("depressive"[All Fields] AND "disorder"[All Fields]) OR "depressive disorder"[All Fields]) AND "after stroke"[Title/Abstract])))  #8 "randomized controlled trial"[Publication Type] OR "controlled clinical trial"[Publication Type] OR "randomized"[Title/Abstract] OR "placebo"[Title/Abstract] OR "randomly"[Title/Abstract] OR "trial"[Title/Abstract] OR "groups"[Title/Abstract]  #9 ("tai chi"[Title/Abstract] OR "taiji*"[Title/Abstract] OR "Qigong"[Title/Abstract] OR "Liuzijue"[Title/Abstract] OR "Wuqinxi"[Title/Abstract] OR "Yijinjing"[Title/Abstract] OR "Baduanjin"[Title/Abstract] OR "traditional exercise"[Title/Abstract] OR "traditional chinese"[Title/Abstract] OR "Medicine"[Title/Abstract] OR "chinese traditional exercise"[Title/Abstract] OR "traditional chinese"[Title/Abstract] OR "chinese exercise"[Title/Abstract] OR ("mindbody"[All Fields] AND "exercise"[Title/Abstract]) OR ("mindbody"[All Fields] AND "Therapies"[Title/Abstract]) OR "mind body therapies"[MeSH Terms]) AND (("post-stroke"[All Fields] AND ("depressive disorder"[MeSH Terms] OR "depression"[MeSH Terms])) OR ("PSD"[Title/Abstract] OR "post stroke depressive"[Title/Abstract] OR "depression after stroke"[Title/Abstract] OR (("depressed"[All Fields] OR "depression"[MeSH Terms] OR "depression"[All Fields] OR "depressions"[All Fields] OR "depression s"[All Fields] OR "depressive disorder"[MeSH Terms] OR ("depressive"[All Fields] AND "disorder"[All Fields]) OR "depressive disorder"[All Fields] OR "depressivity"[All Fields] OR "depressive"[All Fields] OR "depressively"[All Fields] OR "depressiveness"[All Fields] OR "depressives"[All Fields]) AND "in stroke patients"[Title/Abstract]) OR "post stroke depression"[Title/Abstract] OR "post stroke depressive disorder"[Title/Abstract] OR (("depressive disorder"[MeSH Terms] OR ("depressive"[All Fields] AND "disorder"[All Fields]) OR "depressive disorder"[All Fields]) AND "after stroke"[Title/Abstract]))) AND ("randomized controlled trial"[Publication Type] OR "controlled clinical trial"[Publication Type] OR "randomized"[Title/Abstract] OR "placebo"[Title/Abstract] OR "randomly"[Title/Abstract] OR "trial"[Title/Abstract] OR "groups"[Title/Abstract]) | 101 |
| Web of science | 1: TS=(post-stroke depressive OR post-stroke depression OR Post stroke depressive OR Post stroke depression OR depression after stroke OR depression in stroke patients OR post stroke depressive disorder OR depressive disorder after stroke) and Preprint Citation Index (Exclude – Database)  2: TS=(tai chi OR taiji OR Qigong OR Liuzijue OR Wuqinxi OR Yijinjing OR Baduanjin OR traditional exercise OR traditional chinese Medicine OR chinese traditional exercise OR traditional chinese OR chinese exercise OR Mindbody exercise OR MindBody Therapies ) and Preprint Citation Index (Exclude – Database)  3: TS=("random*" OR allocation OR "random allocation" OR placebo OR single blind OR single blind method OR double blind OR double blind method OR "randomized controlled trial*" OR "randomised controlled trial*" OR "RCT" OR "clinical trial*") and Preprint Citation Index (Exclude – Database)  4: #3 AND #2 AND #1 and Preprint Citation Index (Exclude – Database) | 118 |
| Embase | #1. 'post-stroke depression'/exp  #2. 'psd':ti OR 'post-stroke depressive':ti OR 'post-stroke depression':ti OR 'post stroke depressive':ti OR 'post stroke depression':ti OR 'depression after stroke':ti OR 'depression in stroke patients':ti OR 'post stroke depressive disorder':ti OR 'depressive disorder after stroke':ti  #3. 'alternative medicine'/exp OR 'alternative  #4. 'mind-body therapies':ti OR 'tai chi':ti OR 'taiji*':ti OR 'qigong':ti OR 'liuzijue':ti OR 'wuqinxi':ti OR 'yijinjing':ti OR 'baduanjin':ti OR 'traditional exercise':ti OR 'traditional chinese medicine':ti OR 'chinese traditional exercise':ti OR 'traditional chinese':ti OR 'chinese exercise':ti OR 'mindbody exercise':ti OR 'mindbody therapies':ti  #5. 'randomization'/exp OR 'placebo'/exp OR 'placebo effect'/exp OR 'single blind procedure'/exp OR 'double blind procedure'/exp OR 'randomized controlled trial'/exp OR 'randomized controlled trial (topic)'/exp OR 'controlled clinical trial'/exp OR 'controlled clinical trial (topic)'/exp OR 'clinical trial'/exp OR 'clinical trial (topic)'/exp  #6. (((((random*:ab,ti OR allocation:ab,ti OR 'random allocation':ab,ti OR placebo:ab,ti OR single) AND blind:ab,ti OR double) AND blind:ab,ti OR randomised) AND controlled AND trial*:ab,ti OR randomized) AND controlled AND trial*:ab,ti OR  #7. #1 OR #2  #8. #3 OR #4  #9. #5 OR #6  #10. #7 AND #8 AND #9 | 34 |
| Cochrane Library | #1 (random*) or allocation or "random allocation" or placebo or single blind or double blind or (randomized controlled trial*) or RCT or (clinical trial*) 1809793  #2 randomized controlled trial:pt or clinical trial:pt 410455  #3 #1 OR #2 1809793  #4 (PSD):ti,ab,kw OR (post-stroke depressive):ti,ab,kw OR (post-stroke depression):ti,ab,kw OR (Post stroke depressive):ti,ab,kw OR (Post stroke depression):ti,ab,kw OR (depression after stroke):ti,ab,kw OR (depression in stroke patients):ti,ab,kw OR (post stroke depressive disorder):ti,ab,kw OR (depressive disorder after stroke):ti,ab,kw 3717  #5 (tai chi):ti,ab,kw OR (taiji*):ti,ab,kw OR (Qigong):ti,ab,kw OR (Liuzijue):ti,ab,kw OR (Wuqinxi):ti,ab,kw OR (Yijinjing):ti,ab,kw OR (Baduanjin):ti,ab,kw OR (traditional exercise):ti,ab,kw OR (traditional chinese Medicine):ti,ab,kw OR (chinese traditional exercise):ti,ab,kw OR ( traditional chinese):ti,ab,kw OR (chinese exercise):ti,ab,kw OR (Mindbody exercise):ti,ab,kw OR (MindBody Therapies):ti,ab,kw 20368  #6 #3 AND #4 AND #5 151 | 150 |
| CNKI | SU=(太极 + 气功 + 八段锦 + 五禽戏 + 六字诀 + 武术 + 中国传统功法 + 中医运动 + 易筋经 + 功夫 + 站桩 + 形意拳 + 导引术 + 八卦掌) AND SU=卒中后抑郁 | 9 |
| Wanfang | 主题:(太极 OR 气功 OR 八段锦 OR 五禽戏 OR 六字诀 OR 武术 OR 中国传统功法 OR 中医运动 OR 易筋经 OR 功夫 OR 站桩 OR 形意拳 OR 导引术 OR 八卦掌) and 主题:(卒中后抑郁) | 34 |
| VIP | ((M=太极 OR 气功 OR 八段锦 OR 五禽戏 OR 六字诀 OR 武术 OR 中国传统功法 OR 中医运动 OR 易筋经 OR 功夫 OR 站桩 OR 形意拳 OR 导引术 OR 八卦掌) OR (R=太极 OR 气功 OR 八段锦 OR 五禽戏 OR 六字诀 OR 武术 OR 中国传统功法 OR 中医运动 OR 易筋经 OR 功夫 OR 站桩 OR 形意拳 OR 导引术 OR 八卦掌) ) AND ((M=卒中后抑郁) OR (R=卒中后抑郁)) | 35 |
| CBM | (卒中后抑郁) AND (( "太极"[常用字段:智能] OR "气功"[常用字段:智能] OR "八段锦"[常用字段:智能] OR "五禽戏"[常用字段:智能] OR "六字诀"[常用字段:智能] OR "武术"[常用字段:智能] OR "中国传统功法"[常用字段:智能] OR "中医运动"[常用字段:智能] OR "易筋经"[常用字段:智能] OR "功夫"[常用字段:智能] OR "站桩"[常用字段:智能] OR "形意拳"[常用字段:智能] OR "导引术"[常用字段:智能] OR "八卦掌)"[常用字段:智能] OR "(R=太极"[常用字段:智能] OR "气功"[常用字段:智能] OR "八段锦"[常用字段:智能] OR "五禽戏"[常用字段:智能] OR "六字诀"[常用字段:智能] OR "武术"[常用字段:智能] OR "中国传统功法"[常用字段:智能] OR "中医运动"[常用字段:智能] OR "易筋经"[常用字段:智能] OR "功夫"[常用字段:智能] OR "站桩"[常用字段:智能] OR "形意拳"[常用字段:智能] OR "导引术"[常用字段:智能] OR "八卦掌"[常用字段:智能])) | 19 |

Table note:CNKI:China National Knowledge Infrastructure; CBM: China Biology Medicine disc;
