# Supplementary material for: Effects of traditional Chinese exercises on post-stroke depression: a meta-analysis of randomized controlled trials
Source: Front Public Health. 2025 May 12;13:1570878. doi: 10.3389/fpubh.2025.1570878 (PMC12125479; doi:10.3389/fpubh.2025.1570878)
Supplement: Supplementary file 2 [file Image_1.pdf]

Supplementary Appendix Figure 1：Eggers test results of HAMD outcome indicators

.

Number of studies = 10 Root MSE = 1.098

Std_Eff	Coefficient	Std. err.	t	P>|t|	[95% conf.	interval]
slope	3.479886	.690059	5.04	0.001	1.888607	5.071164
bias	-16.64995	2.407525	-6.92	0.000	-22.20172	-11.09819

Test of H0: no small-study effects P = 0.000
